# Granulocytic Myeloid-Derived Suppressor Cells in Cystic Fibrosis

**DOI:** 10.3389/fimmu.2021.745326

**Published:** 2021-09-21

**Authors:** Samantha L. Tucker, Demba Sarr, Balázs Rada

**Affiliations:** Department of Infectious Diseases, College of Veterinary Medicine, The University of Georgia, Athens, GA, United States

**Keywords:** cystic fibrosis, myeloid-derived suppressor cell, neutrophil, immunosuppression, gMDSC

## Abstract

Cystic Fibrosis (CF) is a genetic disease that causes chronic and severe lung inflammation and infection associated with high rates of mortality. In CF, disrupted ion exchange in the epithelium results in excessive mucus production and reduced mucociliary clearance, leading to immune system exacerbation and chronic infections with pathogens such as *P. aeruginosa* and *S. aureus*. Constant immune stimulation leads to altered immune responses including T cell impairment and neutrophil dysfunction. Specifically, CF is considered a Th17-mediated disease, and it has been proposed that both *P. aeruginosa* and a subset of neutrophils known as granulocytic myeloid suppressor cells (gMDSCs) play a role in T cell suppression. The exact mechanisms behind these interactions are yet to be determined, but recent works demonstrate a role for arginase-1. It is also believed that *P. aeruginosa* drives gMDSC function as a means of immune evasion, leading to chronic infection. Herein, we review the current literature regarding immune suppression in CF by gMDSCs with an emphasis on T cell impairment and the role of *P. aeruginosa* in this dynamic interaction.

## Neutrophil Dysfunction in CF

Cystic Fibrosis (CF) is an autosomal recessive disease caused by mutations in the Cystic Fibrosis Transmembrane Conductance Regulator (*CFTR*) gene (1–3). CF is primarily found in the Caucasian population, with an estimated 70,000 individuals affected by the disease (1, 4). Disruption in CFTR function leads to ion dysregulation, abnormal pH, mucus build-up, chronic inflammation, and infection with pathogens such as *Pseudomonas aeruginosa* and *Staphylococcus aureus*. Symptoms in the lungs cause most of the morbidity and mortality in CF (4–13). Neutrophils are major drivers of chronic inflammation in CF airways (14–18). Neutrophils in general are inefficient at pathogen clearance in CF (19–24). In CF and other diseases, it is becoming more evident that different subpopulations of neutrophils exist that may be linked to varying forms of immune dysfunctions (25–30). An increasing body of work exists demonstrating the negative impact of neutrophils on lung disease outcome in CF. Excessive neutrophil recruitment to the lungs leads to increased levels of inflammatory cytokines such as IL-1β, IL-8, IL-17 and IL-6 (6, 31, 32). As neutrophils become activated, damaging granule components such as neutrophil elastase (NE) and metalloproteinase 9 (MMP9) are released into the extracellular space, resulting in perpetuated tissue injury and immune cell recruitment (5, 33, 34). NE has been described to inhibit the function of other cells found in the CF airways (epithelium, macrophages, dendritic cells) and represents a clinically highly relevant target for the pharmaceutical industry (35–39). CF sputum PMN counts, levels of ecDNA, myeloperoxidase (MPO), NE and PMN chemoattractants all correlate with CF lung disease severity (2–6). Phenotypic changes to neutrophils also occur upon entry into the CF airway environment including reduction in surface expression of the phagocytic markers CD16, CD14, and CD35, as well as increased surface expression of activation and degranulation markers CD66b and CD63 (40, 41). Additionally, changes in antigen presentation markers such as CD80, MHCII, and CD294 indicate that CF airway neutrophils potentially interact with T cells (40, 41).

Despite increased neutrophil recruitment to the CF airways, chronic infections with CF-related pathogens such as *P. aeruginosa and Staphylococcus aureus* suggest impairment of neutrophil-mediated killing of these pathogens (19, 21–23, 42). Exacerbated release of neutrophil extracellular traps (NETs) in CF airways (19, 43, 44), as well as increased NET formation in response to clinical isolates of *P. aeruginosa* from CF patients have been observed (17, 19, 20, 45–47). Another study demonstrated increased TLR5 surface expression on CF airway neutrophils compared to CF blood neutrophils and blood and airway neutrophils from healthy and non-CF bronchiectasis donors (48). This work further demonstrated that incubation of blood neutrophils in CF sputum supernatant increased TLR5 surface expression (48). It was previously shown that NETs represent a main mechanism of *P. aeruginosa* killing by neutrophils in *in vitro* suspension co-cultures (19). Mucoid *P. aeruginosa* was shown to be resistant to neutrophil-mediated killing (19, 46). Overall, these data suggest that antimicrobial effector functions of neutrophils are impaired in CF that could be due to enhanced immunosuppressive functions of the cells.

## MDSCs

**Figure 1:**
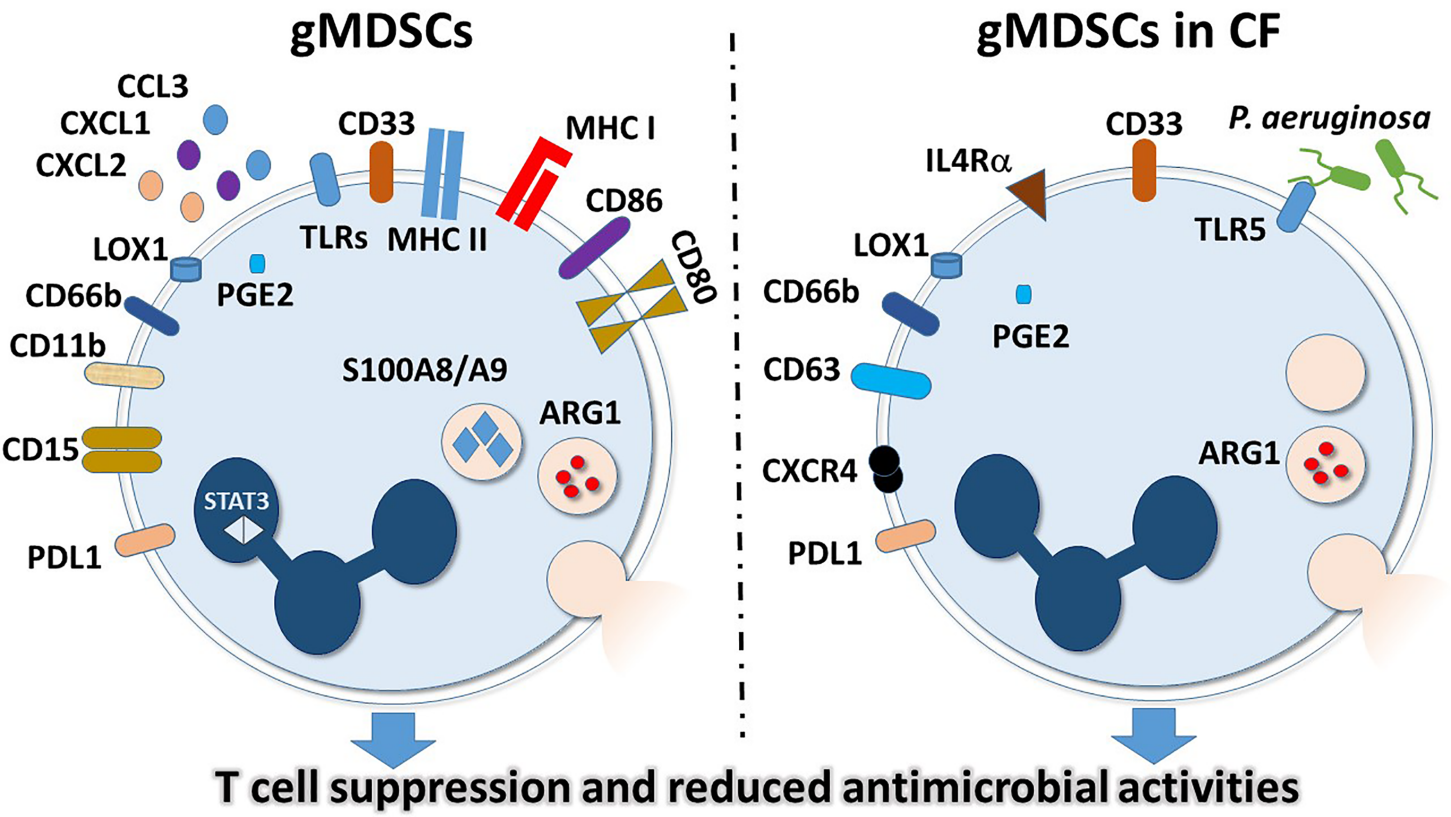
General and CF-specific features of gMDSCs. Surface markers and intracellular molecules are indicated that have higher expressions or activities in granulocytic MDSCs in general (left panel) or in gMDSCs in CF (right panel).

Immunosuppressive myeloid cells have been first described about three decades ago. While several names were proposed, in 2007 the term ‘Myeloid-derived suppressor cells (MDSCs)’ was coined to identify monocytes and neutrophils with powerful immunosuppressive features (49, 50). MDSCs have been mainly linked to pathological conditions in cancer, inflammation and autoimmune disease and their physiological roles have also been described (51). In general, two types of MDSCs have been distinguished: monocytic MDSCs (mMDSCs, M-MDSCs) and granulocytic/polymorphonuclear MDSCs (gMDSCs, also abbreviated as G-MDSCs or PMN-MDSCs) (51). There are several reviews that summarize current knowledge on MDSCs and their detailed role in diseases (51). The purpose of this review is to provide a brief summary and introduction to MDSCs and to specifically summarize the proposed roles of gMDSCs in CF only (**Figure 1**). Even though MDSCs have been studied for years, their origin and development remain largely unclear (51). A consensus among scientists exists related to the development of MDSCs from myeloid cells that are in an immature state (51). MDSCs are primarily defined by their immunosuppressive function and myeloid origin, and do not represent a well-defined, single cell subset (51). This is also reflected by the fact that cell surface markers specific to MDSCs that have been widely accepted by the scientific community have not been identified yet.

Neutrophils were originally thought to be terminally differentiated, proinflammatory cells, only responsible for and capable of pathogen elimination. However, it has recently become apparent that neutrophils represent a heterogeneous population that differ in maturity, density and inflammatory properties (25–28, 30, 52). The heterogeneity of neutrophils was first discovered in cancer patients, where a portion of neutrophils co-purified in the mononuclear cell fraction during peripheral blood cell isolation (53). In this study, it was determined that these lower density neutrophils (LDNs) were activated, less dense than normal neutrophils and capable of suppressing T cell signaling in a hydrogen peroxide-dependent manner (53). Because of the suppressor phenotype of these LDNs, the term granulocytic myeloid-derived suppressor cells (gMDSCs, also called PMN-MDSCs) was coined (53). gMDSCs describe a subset of myeloid cells expressing neutrophilic markers and are characterized by an immunosuppressive phenotype. This is in contrast to monocyte-derived MDSCs, which have similar functions, but stem from a different, monocytic lineage (51). Additional works demonstrating TGF-β-modulated polarization of protumor and anti-tumor neutrophils added to the clear presence of multiple neutrophil types (26). Specifically, blockage of TGF-β signaling resulted in increased cytotoxic, hypersegmented anti-tumor neutrophils (N1), whereas the presence of TGF-β resulted in less aggressive protumor neutrophils (N2) (26). Transcriptomic analyses in mice examined normal neutrophils from healthy animals, tumor-associated neutrophils (TANs) and splenic gMDSCs from cancer-positive animals distinguished the phenotypic differences of these cells (27). It was shown that while normal neutrophils and gMDSCs likely come from the same progenitors, they have very different mRNA profiles. Specifically, gMDSCs are primed for antigen presentation and highly express MHC class I and II as well as the co-stimulatory molecules CD80 and CD86 (27) (**Figure 1**). Enhanced antigen-presenting capacity is in line with data demonstrating gMDSC interactions with T cells (53). Additional changes in gMDSCs included increased expressions of TLRs and BCL-2-related apoptotic genes (27). Lastly, the expressions of neutrophil chemoattractants CXCL1, CXCL2 and CCL3 were markedly higher in gMDSCs compared to normal neutrophils (27). These studies highlight the phenotypic variability that occurs among neutrophils within an individual (**Figure 1**).

In the past decade, multiple studies have come out addressing additional differences between normal neutrophils and gMDSCs, and brought more questions than answers. For example, while gMDSCs were originally considered immunosuppressive LDNs and co-purified by density centrifugation with mononuclear cells in cancer patients, other LDNs have been found in autoimmune diseases such as systemic lupus erythematosus (SLE), are known to be hyper-inflammatory and cause vascular damage (25, 52, 54). It has recently been determined that the presence or absence of CD10 determines the maturity status of LDNs, and can distinguish between mature cells (CD10^+^) which have an immunosuppressive phenotype, and immature cells (CD10^-^) with an immune-stimulatory phenotype (28, 54). Maturation resulting in CD10 expression and immune suppression appears to be driven by G-CSF (28).

Numerous reviews exist describing the current literature available on the heterogeneity of neutrophils as well as gMDSCs and LDGs (29, 30, 51). The currently accepted characterization for gMDSCs isolated from human peripheral blood describes these cells as low-density neutrophils expressing CD11b, CD15, CD66b, LOX-1, and lacking CD14 (51, 53, 55). In mice, gMDSCs are defined as CD11b+ Ly6G+ while mMDCS are CD11b+ Ly6C+, the same way by which mature neutrophils and monocytes are determined. Additionally, gMDSCs suppress T cell proliferation as a functional marker, and have very high reactive oxygen species (ROS), ARG-1, PGE2, S100A8/A9, and STAT3 activities, as well as high levels of ER stress (51, 55, 56). Differences in signaling including strength, duration, and major pathway play pivotal roles in the abundance and function of gMDSCs within an individual. It is evident that chronic conditions such as cancer, pregnancy, obesity, or persistent infection lead to a sustained, low level immune response (52). This constant, weak stimulation results in the increased presence of gMDSCs that have reduced phagocytosis, increased ROS production, and are capable of suppressing T cells (17, 18, 51, 55, 57–59). Therefore, in comparison to normal neutrophils, gMDSCs seem to be reducing inflammation and cease the perpetual signaling that results from chronic immune stimulation (**Figure 1**).

## gMDSCs in Cystic Fibrosis

It remains unclear in chronic diseases whether gMDSCs are only generated in the bone marrow alongside normal neutrophils, or if normal neutrophils can also develop into gMDSCs or gMDSC-like cells at the site of inflammation. One study demonstrated that ER stress leading to the upregulation of LOX-1 expression resulted in neutrophils with gene expression patterns and suppressive capabilities similar to gMDSCs (55). These data suggest that suppressive actions of neutrophils are possibly inducible; however, this has not been examined in CF. In the case of CF, reports of gMDSCs are conflicting with regards to phenotypic differences between normal neutrophils, gMDSCs from peripheral blood, and neutrophils isolated from the lungs (16–18).

Although the initial cause of CF is genetic, the symptoms of reduced ASL and excessive mucus production ultimately result in immune cell recruitment, tissue damage and perpetuated inflammation, which is further exacerbated by chronic bacterial and fungal infections (1, 13, 60, 61). As previously mentioned, neutrophils are abundantly present in CF airways, but fail to clear certain pathogens. This leads to the hypothesis that neutrophils are playing an alternate, immunosuppressive role in the CF airways. Given that *P. aeruginosa* induces T cell suppression as well as TLR5 expression in neutrophils, it was initially proposed that *P. aeruginosa* induces gMDSC production in CF as a means to evade the T cell immune response (16). To this end, it was demonstrated that gMDSCs, identified as CD33^high^/CD66b^high^/IL-4Rα^inter^/HLA-DR^dim^ populations in the PBMC fraction were higher in CF patients compared to healthy controls (16). More importantly, gMDSCs in the PBMC fraction were higher in *P. aeruginosa*-positive individuals compared to *P. aeruginosa*-negative individuals (16). Additionally, while there was no correlation between blood gMDSCs and lung function of *P. aeruginosa-*negative individuals, the number of gMDSCs positively correlated with lung function of *P. aeruginosa-*positive individuals (16). It was further demonstrated that *in vitro* incubation of PBMCs with *P. aeruginosa*, or its flagellin alone induces gMDSCs that highly express TLR5 and CXCR4 in a CFTR-independent manner (16). Lastly, this report demonstrated that both CF-gMDSCs and *in vitro P.aeruginosa*-induced gMDSCs suppress CD4^+^ and CD8^+^ T cell proliferation, as well as IL-17 secretion (16). This study was the first to link gMDSCs to CF disease pathogenesis, and to suggest that in response to prolonged inflammation and infection, gMDSCs may be playing an anti-inflammatory role of reducing T cell proliferation, recruitment of other proinflammatory cells and tissue damage in response to *P. aeruginosa* infection (16) (**Figure 1**).

The report by Rieber et al. defined a function for gMDSCs circulating in the blood; however, to truly understand the role of these cells in CF, it is imperative to assess samples from the lungs and broncheoalveolar lavage fluid (BAL) (16). It has been demonstrated that gMDSCs can suppress T cells through the actions of Programmed Death Ligand 1 (PD-L1), arginase-1 (Arg-1) and ROS (53, 55, 57, 59, 62–66). PD-L1-mediated suppression, by interaction with PD-1, results in activated T cell exhaustion and blockade of secondary signals for activation (57, 59, 66). Arg-1 suppresses T cells by competitively binding arginine and generating L-ornithine (62). The lack of arginine prevents the expression of the ζ-chain of the T cell receptor (TCR) complex and therefore inhibits T cell function (62). To determine the mechanism of T cell suppression by CF-gMDSCs, a study was performed measuring PD-L1 and Arg-1 in both the blood and airways of CF patients (17). Here it was shown that mature airway neutrophils, defined as CD66b^+^/CD63^+^/CXCR4^+^/CD62L^lo^ suppress T cell proliferation through the action of Arg-1, but not PD-L1 (17). Specifically, Arg-1 activity was shown to be higher in CF airway neutrophils compared to healthy controls. Additionally, incubation of PBMCs with CF airway supernatant resulted in reduced T cell proliferation that could be inhibited by a combination treatment with excess arginine and arginase inhibitor, but not by blockage of PD-L1 (17). Lastly, Arg-1 activity positively correlated with total airway neutrophils and negatively correlated with lung function (17). Interestingly, a more recent study determined that mMDSCs isolated from CF patients, characterized as CD14^+^ cells inhibited T cells in a PD-L1-dependent manner (67), suggesting that additional mechanisms exist for immune disruption in CF. Although this work clearly demonstrated the suppressive capabilities of CF airway neutrophils, it did not definitively conclude that these cells represent airway gMDSCs. That being said, the population isolated had many features of gMDSCs from peripheral blood, including CXCR4 expression, Arg-1 activity, and T cell suppression, suggesting the presence of gMDSCs or gMDSC-like cells in the CF airways (17) (**Figure 1**).

The data available on gMDSCs in CF airway disease suggest contributions from both host-driven responses as well as *P. aeruginosa*-mediated responses (16, 17, 40). To further investigate the impact of gMDSCs in the CF airway in regards to *P. aeruginosa* infection, animal studies using *cftr-*deficient mice were performed (18). The number and percent of gMDSCs, defined as CD11b^+^/Ly6C^inter^/Ly6G^high^ cells as well as that of mMDSCs CD11b^+^/Ly6C^high^/Ly6G^-^ were measured in the BAL, lungs, bone marrow, and spleens of c*ftr*-deficient mice with or without *P. aeruginosa* infection (18). It was shown that *P. aeruginosa* infection recruits gMDSCs capable of T cell suppression to the lungs and BAL of *cftr*-deficient mice (18). In contrast, more gMDSCs were present in the bone marrow of uninfected mice, compared to infected animals (18). While a similar trend was noted for mMDSCs in the lung, they were present at a much lower percent compared to gMDSCs (18). To further understand the role of *P. aeruginosa* in gMDSCs’ suppressor activity, gMDSCs were isolated from the lung, spleen, and bone marrow of infected wild-type mice and co-cultured with T cells *in vitro*. This experiment demonstrated that gMDSCs from the lung and bone marrow were both capable of suppressing T cell proliferation (18). Lastly, this paper examined the role of *cftr* in gMDSC function, and showed a slight impairment of T cell suppression in *cftr^-/-^* gMDSCs; however, this impairment only occurred at very high gMDSC to T cell ratios, suggesting that c*ftr* is only minimally involved in T cell suppression by gMDSCs (18, 19). Overall, this study demonstrates that gMDSCs are intrinsic to a CF mouse model, but that *P. aeruginosa* infection is also involved in gMDSC recruitment to the lungs and T cell suppression (18).

## T Cell Function in Cystic Fibrosis

Several reports demonstrate altered T cell responses in CF, with a bias towards Th17 cell production and activity that has been linked to and could be mediated by gMDSCs (14, 15). Th17 cells, IL-17, and other Th17-associated cytokines have been shown to be increased in the BAL of patients with CF (15). The same study reported an association between high IL-17 levels in the BAL and a greater chance of developing *P. aeruginosa* infection within 2 years’ time (15). A negative correlation between lung function (FEV_1_%) and the number of Th17 cells in the peripheral blood has also been demonstrated in CF, suggesting that an increased Th17 response is associated with poorer disease outcome (68). Disruption of regulatory T cells (Tregs) has also been reported in CF (14). Specifically, the percent of Tregs compared to other cell populations was significantly lower in the peripheral blood and BAL of CF patients compared to healthy controls and non-CF bronchiectasis controls (14). It was also demonstrated that patients with chronic *P. aeruginosa* infection had even further reduced amounts of Tregs. These data were confirmed with *cftr* ^-/-^ mouse studies, showing decreased Treg numbers in the spleen and lung, as well as a further reduction in Tregs upon *P. aeruginosa* infection (14). This Treg disruption was not correlated with any other CF-associated pathogen (14). To further confirm the disruption of Tregs in CF, Hector et al. showed that both CFTR inhibitors, as well as incubation with clinically relevant *P. aeruginosa* reduced the percent of Tregs in the peripheral blood isolated from healthy individuals, and that Tregs isolated from CF patients were less suppressory than Tregs from healthy donors (14). This reduction in suppression was further enhanced in CF patients with chronic *P. aeruginosa* infection (14). Finally, this study analyzed Tregs and memory Tregs as a function of age and demonstrated a decline in CF Tregs with age that was increased by chronic *P. aeruginosa* infection, as well as a reduced capacity for generating memory Tregs in these individuals (14). Taken together, these studies demonstrate an impaired adaptive immune response in CF (**Figure 1**).

## Conclusions

Immune system dysregulation is a driving force in CF disease progression and morbidity. Specifically, neutrophils in the lungs are inefficient killers and contribute to tissue damage, inflammation, and chronic infection. Additionally, gMDSCs or gMDSC-like neutrophils could mediate T cell suppression in CF. Suppression of T cells can result in systemic immune system disruption. This interaction between gMDSCs and T cells is further complicated by *P. aeruginosa* infection, which enhances the T cell suppressor phenotype of these neutrophils, and may enhance immune evasion by these bacteria (**Figure 1**). Although additional investigation is needed to fully elucidate how the dynamic relationship between *P. aeruginosa*, gMDSCs, and T cells impact disease exacerbation in CF; these interactions may serve as therapeutic targets for immune dysregulation. Future research into the impact of gMDSCs on T cells and other immune responses will help to determine the multifunctional capacity of neutrophils in CF as well as other chronic inflammatory and infectious diseases.

## Author Contributions

ST conceptualized the idea, wrote and revised the manuscript. DS reviewed the manuscript. BR conceptualized the idea, obtained funding and revised the manuscript. All authors contributed to the article and approved the submitted version.

## Funding

This work was supported by the NIH grant R01HL136707 (to BR).

## Author Disclaimer

The content is solely the responsibility of the authors and does not necessarily represent the official views of the National Institutes of Health.

## Conflict of Interest

The authors declare that the research was conducted in the absence of any commercial or financial relationships that could be construed as a potential conflict of interest.

## Publisher’s Note

All claims expressed in this article are solely those of the authors and do not necessarily represent those of their affiliated organizations, or those of the publisher, the editors and the reviewers. Any product that may be evaluated in this article, or claim that may be made by its manufacturer, is not guaranteed or endorsed by the publisher.
